# Philadelphia Hospital

**Published:** 1838-03-14

**Authors:** 


### PHILADELPHIA HOSPITAL.

### EPILEPSY.

*Saturday, 10th February.* Dr. Jackson commenced: Epilepsy, gentlemen, is one of the most formidable affections, which you will be called upon to treat; and you will find few, in which all the resources of your art will so frequently fail. It has been looked upon in this light, from the earliest period. The ancients had so little control over it, that they designated it by a term signifying the malice of the demon. Hippocrates continued the title *morbus sacer*, or the sacred disease, though he combated the superstitions from which it was derived. Various other terms have been applied to it. By the Romans, it was called *morbus comitialis*, from the fact that its victims were often struck down with it in the forum, probably owing to the excitement arising out of public discussions, and political warmth.

Many illustrious and eminent characters of ancient and modern times have been subject to this infirmity. Julius Cæsar* was an epileptic sufferer: it is very certain, therefore, that it is a disease, which does not necessarily interfere with a high development and exercise of the intellectual faculties. On the contrary, this very exercise of them is often productive of the disease. Perhaps the most distinguished pulpit orator this country has produced, Buckminster, fell a victim to repeated

* *Casca.* He fell down in the market place, and foamed at the mouth, and was speechless.

*Brutus.* 'Tis very like: he hath the falling sickness.

*Julius Cæsar. Act 1st, scene 2d.*

attacks of this affection, brought on by intense mental application. On the other hand, fatuity is not an unfrequent attendant upon epilepsy, as you will have occasion to notice, in numerous specimens of the disease, that will shortly be presented to you.

Epilepsy is vulgarly called the falling sickness, from the circumstance of the patient's falling suddenly to the ground, upon an attack of it. From this cause, they are liable to numerous accidents; in several in this house, that will be brought before you, you will see traces of the effects of fire, and other injuries.

Epilepsy is a convulsive affection, attended with loss of consciousness. This latter feature I am anxious to impress particularly upon your attention, as it is not considered by some writers, among them Vogel, Sauvages, and even Cullen, as essentially characteristic of the disorder. For my part, I never saw a case, certainly never in this house, in which it was wanting. The symptoms of epilepsy then I consider to be, convulsive movements, with loss of consciousness and of the intellectual faculties. As the loss of the powers of motion in palsy depends upon pressure acting upon the thalamus and corpus striatum, so I think we have evidence from the convulsions of epilepsy, combined with loss of consciousness, that in it, the cerebral structure must be involved. But the convulsions I do not look upon as the most important symptom; that I consider to be the loss of the intellectual faculties—the muscular symptoms being the first to pass off, and frequently very slight. In some cases, the muscular convulsions and loss of the faculties are followed by complete coma. The suspension of the intellectual faculties, and the convulsive movements are evidently the effects of sudden and violent disorder in the cerebral circulation. There is, it appears to me, a raptus of the capillary circulation, terminating in a congestion, more or less intense, of the brain. This is usually well marked, the face swelling up, and becoming turgid, and almost purple, affording striking evidence of determination of blood to the head. Occasionally, so forcible is this raptus, that ecchymosis occurs from effusion of the blood out of its vessels. This fact, it strikes me, is strong evidence, that the movement is attended with force, is one of activity, and that the accumulation of blood, as has been conjectured, is passive and secondary. I had an illustration of this fact, in the case of a gentleman, who was under my care some years ago, in whom the disease suddenly showed itself. Attention was first called to him, by his family being struck with the appearance of petechiæ, or livid spots upon his face. A doctor was sent for, who pronounced it a mere affection of the skin, which would disappear spontaneously, as in fact it did; but, in a week or two, it came back again; and this continued to be repeated for some months, without a suspicion of its cause, or of the true nature of the disease. A young man, for some accidental reason, happened to sleep in the same bed with him, and was awakened in the night, from his agitation in a convulsion. It was then ascertained, that the periodical appearance of spots on the face proceeded from attacks of epilepsy in the night, of which the patient was unconscious.

The patient, in this affection, is totally unconscious of the existence of an attack. He rouses up gradually from a state of profound somnolency, with an expression of astonishment upon his countenance, with simply an unusual feeling of soreness, from the great muscular exertion which he has undergone. This, to an epileptic, is the principal indication that he has had an attack.

The attack varies in character. Sometimes the patient falls suddenly down, without any previous admonition. At other times, he is able to anticipate the approach of the complaint. Most frequently it is preceded, by an uncomfortable sensation about the head, as vertigo, dimness of sight, &c. Again, the warning may take place in the abdominal viscera, which is ordinarily indicated by a sensation about the region of the stomach, gradually moving upwards to the head. Often, however, the patient has no consciousness of his approaching fate; he suddenly cries out and falls prostrate to the ground.

The symptoms of an attack are not always of this extreme intensity. Sometimes they are so mild that you can hardly call them epilepsy, though I think they still belong to this class of affections. They are often exceedingly local in their character. A gentleman of the medical class, some years ago, was affected in this manner. He had been recommended to study medicine, as a mode of getting rid of the disorder, which, by the way, was a very ill-judged recommendation. He would at times in conversation, suddenly repeat over some unconnected words, or an oath, or he would sing a little, being all the while in a state of total unconsciousness of what he was doing, and then resume the thread of his discourse, ignorant, unless informed of it, that any thing had occurred.

The disease was limited to the production of the above symptoms, except on one occasion, when he imprudently fatigued himself with exertion, and ate a lage quantity of nuts, (a quart.) He then had a complete epileptic convulsion. This accident indicated the true character of the disease.

Another case, in which the disease showed itself in this slight local form, was that of a gentleman, who had a daughter also labouring under the disease of a most strongly marked character. I have often seen this gentleman stop, while in conversation, and repeat his words; sometimes he would merely move his lips without articulating, and then resume his discourse, without being conscious of what had happened.

I have met with one case that appeared to me, if it may be so called, epilepsy affecting the spinal marrow. The general character of the symptoms evidently connect it with epilepsy. This case was that of a boy, seven or eight years of age, who laboured under monthly attacks, of a form which I shall presently describe, brought on by an obstinate intermittent fever, when an infant. Epilepsy supervened, and had continued.

These attacks were also of an intermittent character; indeed, I might here mention that the disease always takes on a type more or less intermittent.

The paroxysms would continue to recur for three days, and often amounted to twenty or thirty, in the twenty-four hours. At the end of three days, they disappeared, until the expiration of a month, when they would be again renewed. In all other respects his health was perfect. He was seized every month, without any loss of consciousness, however, with spasms, affecting all the muscles of the trunk and extremities, proceeding of course from an affection of the spinal marrow. It was a case of cramps, more than of convulsions. They were attended, as all cramps are, with violent pains.

This boy is now fourteen years of age, and with the appearance of puberty, the affection is disappearing. I cannot well rank this affection any where in a system of pathology. It is not epilepsy, because the brain is not affected, nor can it be called tetanus. I think I may perhaps with propriety, designate it epilepsy of the spinal marrow.

After this general outline of the symptoms and character of the disease, I will now introduce to your notice some cases of patients who are labouring under it. I will only add previously, that the most common period for it to show itself is childhood, and that it is more apt to attack the female than the male sex.

This girl is twenty-two years of age, and enjoys general good health. You notice the vacant expression of her countenance. There is idiotism combined with epilepsy: this is not unfrequent. She menstruates irregularly; the menstrual function was not established, till three years since; the secretion is now scanty, but the discharge occurs more or less every month. Epileptic fits first appeared seven years ago, when she was fifteen years of age; up to that time, she had been quite well. At first, they were periodical, recurring every three weeks; subsequently, after the menstrual evacuation was established, they became more and more frequent. At present, epileptic paroxysms precede and accompany the menstrual period. The patient is sensible of the approach of the fits, by the sensation of a bright light before the left eye which precedes the attack: during the attack, she is insensible. All the functions are performed with regularity except the menstrual. The girl has never received any external injury. You learn that she has a premonition of the approach of an attack. This is often the case. The patient usually feels a rising up from the epigastrium to the head, like a flame, termed the aura epileptica. When it has reached the head, consciousness is gone. This girl tells me that her attacks correspond with changes of the moon. It has been a favourite theory with many writers, that lunar influence is felt by individuals, suffering under this and other affections. Balfour has written a work in favour of this hypothesis. I however have no sort of belief in any connection between epilepsy and changes of the moon. In this case, from the intermittent character of the affection, it occurring every three or four weeks, the attacks must correspond in many cases, with some one of the four quarters of the moon.

In this case, the appetite is normal. It is sometimes exceedingly voracious, and cases of this sort I never could cure: I believe them to be totally incurable. A regulation of the diet is a most important element, in the successful treatment of epilepsy; and, as this morbid appetite prevents any control over the diet, a cure is hopeless.

The memory in this girl is impaired. I have gathered however from her that she has dysmenorrhea, and menstruates with pain. I speak thus freely before her, as the faculties of her mind are impaired. Delicacy on this subject to the sex, gentlemen, should never be out of mind. The uterus then, in this case, I consider the seat of the disease, the point from which morbid action is reflected to the brain. Nutrition is, you see, not affected by the disease: this girl's flesh is firm, and her digestive powers appear to be in excellent condition. The cerebro-spinal system may be affected, without impairing the constitution; and you see how the life of nutrition is independent of the brain itself.

Here is another case, an old inmate of this house, a girl aged 26, of very obtuse intelligence; although you see she recollects my face and name. Notwithstanding this great pallor of countenance, which you notice, she enjoys pretty good general health. She menstruates regularly though with some slight pain. The first fit of the disease occured at the age of eighteen. She was attacked suddenly whilst engaged at her ordinary occupation, and does not recollect that she had any pain in the head or unpleasant feelings about that time: she says she understands that she had not. At first the fits occurred almost daily. Her attacks now are usually preceded at night by fulness of the head, and a state of insensibility, attended by a blackened turgid face, but without convulsive movements: these are succeeded the next morning by regular fits of epilepsy, to the number of fifteen, during the twenty-four hours. Now these previous nightly premonitory attacks were called fainting-fits, but they are quite the contrary. Fainting takes place from want of action in the heart; the blood does *not* go to the brain, and you have feeble pulse and respiration and *pallid face*. Whereas, here, you have evident determination of blood to the head, full pulse, and quick respiration.

This third case presents an example of the voracious appetite of which I spoke. She is twenty years of age, and has been subject to the disease from the age of five. Her mental imbecility is very great. Menstruation regular. The paroxysms are of daily occurrence; during them, her face becomes exceedingly flushed, and she froths at the mouth. Some time ago, she says, she felt queer when the fit was coming on, but she has now no admonition of its approach.

Here is another case, in which the disease is of seventeen years standing, and showed itself first when the woman was between sixteen and seventeen years of age. It has proved very intractable, every description of treatment having failed to make an impression upon it. The intelligence, however, as you perceive remains very perfect. The paroxysms are preceded by dysmenorrhea, which gives the affection an uterine type.

Here is a case, the general character of which does not differ from the others, which have been under your notice, except that the disease first appeared after an attack of scarlatina, in which the head was much affected. The girl is sixteen years of age, and has never menstruated. You see the total imbecility of her mind; her memory is gone. The nurse tells us, that the attack of scarlatina occurred five years since, and that her mind was perfect before. The paroxysms of epilepsy are now becoming more frequent, occurring almost daily.

They come on now at night, although formerly she had them in the day time. When on the history of the disease, I should have mentioned, that many individuals have the fits only at night, and never during the day, the only manifestation of the occurrence of the attack, being a soreness in the muscles next morning.

You have next before you a man, aged forty-seven years, in whom the disease has existed for thirteen years, caused by a blow on the head from the fall of a tree. Several pieces of bone were taken out at the time, and the wound had nearly healed, when he had the first paroxysm of epilepsy, about three months after the injury. At first the paroxysms were very severe: he thinks they now grow lighter. They were more severe when he was in the habit of taking his dram. He says they are now brought on by his "taking cold," and that he never has them when his health is perfectly regular. You see then that the disease is clearly here under the influence of exciting causes. The last paroxysm the man had was about three months ago. His memory is much impaired, as to events which have transpired since the injury; but he can recollect well whatever took place before.

I have met with many such cases as this, in which the memory totally failed in events subsequent to the appearance of the disease, although it retained perfectly facts antecedent to this epoch. I had some years ago a boy from Virginia under my care, a strong, vigorous lad, in whom epilepsy had been brought on from excessive mental application. He was a very ambitious little fellow, always striving to be at the head of his class, and his tutor, instead of repressing, injudiciously urged on his efforts. His recollection of things that had happened previous to his attack was perfect, and of all that he had learned, but he was incapable of acquiring or retaining an idea subsequently. I took him home to live with me, and I was never able to communicate to him a new idea. I could not even make him understand that the Virginia penny and our cent were the same coin.

I perceive that the man, of whom we were just now speaking, has a depression of the bones of his skull of a quarter of an inch. This may be the local cause of the affection. I may add, as regards this case, that the man knows when an attack is coming on, by the appearance of a black spot before his right eye, three or four minutes before he falls. He always lies down, when he finds the fit coming on, and it is never so sudden as to prevent this.

I present to you another man, aged twenty-seven, in whom the disease has existed say for ten years. It was brought about by excessive intemperance. Here then you perceive another exciting cause of the disease. You observe that in this man, the muscular movement is affected. This is by no means necessarily the case. All the others you saw walked well. On the contrary, I have generally seen considerable muscular development and muscular power in epileptic patients.

Here is a case complicated with mania, the general health being otherwise excellent, another illustration how little the functions of nutrition are affected in the diseases of the cerebral organs. I have here another case complicated with mania, in which the disease has existed for three years. The patient was scalded in a steamboat, just before the epilepsy appeared: this may probably have had some connection with the production of it.

In this boy, whom I now show you, the paroxysm often manifests itself with simple loss of muscular power. The boy, I am told, will fall without any accompanying loss of consciousness. In some cases, there is merely a slight vertiginous movement, the patient not losing his consciousness and instantly recovering: the paroxysm is not then complete. It is a case of incomplete epilepsy.

The train of cases, which I have exhibited to you, affords a melancholy proof of the inefficiency of all the resources of art and science. Epilepsy appears to have the same incorrigible character now, as at the earliest periods.

These cases have shown a succession of exciting causes of the disease. I shall not however divide it into different species, according to the local origin that makes an impression on the brain. The essential character of the disease I hold to be a highly irritable erythematic state of the brain, which if you strike away, you have various local affections, but no longer epilepsy. By analysing the several distinct movements, which take place in the animal economy, in the affection, I think we may found a rational pathology of the disease and system of treatment. The treatment of epilepsy, as generally directed, is empirical. There is no one remedy upon which we can rely, but we must adapt our therapeutics to the features of the case. These may be as various as the causes of the disease.

By analysis, then, I think we will find three perfectly distinct features in epilepsy. The first is an irritable state of the cerebral structure—possibly an erythematic condition of the brain. This condition of the brain proceeds from various causes. It may be occasioned by some local affection of a painful or irritative character, with which the brain is ultimately brought into sympathy, or, it may be induced by a variety of exciting causes, such as excess of venereal indulgence, masturbation, exostosis of the cranium, spicula of bone or tumours upon the arachnoid. You readily see, how these latter must keep up a constant state of irritation within the cranium. The race of Indians, who flatten the head by artificial means, and force in the bone upon the brain, are said to be epileptic. Cases of exostosis, spicula, and depression of the bone are difficult of management, though I will not say they are utterly intractable. Professor Dudley recommends the trephine, in such instances, and he has been remarkably successful, in relieving some cases by this operation. Winter before last, Professor Gibson performed an operation of this sort, in this hospital, in a case, where depression existed, caused by an accident, but without beneficial result. This case appeared to me to promise a favourable opportunity for testing the influence of this proceeding: it did not even suspend the paroxysms.

The next element, which I consider a component of the disease, is, the extreme mobility of the capillary circulation, and the facility with which its' regular distribution is deranged. If you prevent this, you prevent the formation of the paroxysm. The essential feature of the disease is, a sudden congestion of blood on the brain, apparently produced by a raptus or rush on that organ. The symptoms that result are dependent upon the intensity of this raptus. In some cases, it is so violent, that petechial effusion of blood in the skin of the face occurs; and the attack varies from a slight loss of consciousness, and a light convulsion, succeeded by a transient sleep, to prolonged and profound coma, that will last for two or three hours.

The next and last element is, the existence of some permanent local affection, which acts as a thorn in the flesh, or point of irritation, worrying the nervous system into excitement, and producing an irritable or erythematic state of the brain. This may be, as you saw in many of the cases to-day before you, dysmenorrhea, or other uterine disease. When on the subject of neuralgia, I told you, that these might act as excitants of that disease.

Another local cause of epilepsy is intestinal irritation. The existence of worms has frequently been the exciting cause. Your remedies then are anthelmintics: you purge with turpentine or someting of the kind, and cure the patient. Derangement of the stomach may also act as a local cause of irritation upon the brain. Ordinary convulsions are, you know, often produced by the functions of digestion being impaired: this sort of reflex action may take place through the medium either of the ganglionic system, or of the eighth pair of nerves. I could recall several instances of fatal convulsions from the action of indigestible food. Such a one occurred in my practice, in the case of a young child, two years of age, from a large quantity of fried eggs. It was destroyed in four days. You perceive here, that a simple article of food may produce convulsions, a violent congestion, or other disorder of the brain, from a cause that can act only on the stomach. You can understand, then, how a morbid state of the stomach, reflected upon the brain, may produce a state, productive of epiletic paroxysms. The congestive raptus, the immediate cause of the convulsion and unconsciousness takes place by a sort of *appel*, if I may use the word, upon the grand capillary circulation, which is the means of the circulatory communication between the organ locally affected and the brain.

These then, gentlemen, are my views of the pathology of epilepsy. I do not look upon it as a single disease, in which there is but one element, but as consisting of several combined elements: first, as erythematic irritability of the brain, secondly, a morbid mobility, through the action of the nervous system, of the capillary circulation by which its equal distribution is easily disturbed; and thirdly, a local affection, a point of irritation in some distant organ, which, by a reflex action upon the brain, excites and keeps up the disease. If these views are correct,—and, if you analyse the phenomena of the disease with care, I think you will find it resolves itself into the elements I have enumerated, we have at once a rational plan of treatment, based upon the different component parts of the affection. For each element, we must have a different mode of treatment. Our efforts must be directed in the first place, to get rid of the erythematic or irritable state of the brain, secondly, to give tone to the capillary system, and, thirdly, to remove the local cause of the disease.

The first thing to be done is, to place your patient upon the lightest possible diet, so as to have the stomach completely under your control. Of course alcoholic liquors, tea, coffee, and the stimulants generally are to be utterly prohibited. As a general rule, the food is to be exclusively vegetable. I have never known a case to recover, when animal food was continued. We then direct our treatment to the brain. I commence by having the hair cut short, and by a course of chronic leeching. One day, I apply a leech to one ear, then another to the temple, a third day behind the other ear, and so on. This I keep up for months: in fact, you must prepare your patient for a long period of probation, holding out no prospect of relief, before one or two years have elapsed. Cold water is to be applied to the head, two or three times every day, with at the same time, warm or stimulating foot-baths. When the patient is asleep, keep warm bricks or warm water constantly to the feet. He is besides to sleep upon a hard pillow, without a night-cap, upon a mattress and not a feather-bed. All this is to keep down cerebral excitement and prevent the flow of blood to the head. Setons at the back of the neck, or an issue on the arm, as a diverticulum, are useful. Your next object is, to give security to the capillary system. For this purpose, you must employ tonics, of which the best for your purpose are the mineral tonics. I prefer the preparations of zinc. Begin with a quarter of a grain of the sulphate of zinc and one grain of the oxide, two or three times a day, and gradually increase the sulphate to one grain. Emetics have been recommended and tartar emetic has been employed, just before the paroxysm is about to occur: if you can foresee this, this treatment may be of advantage. But I prefer to vomit with the sulphate of zinc. By persisting with the use of the metallic preparations, the capillary system gradually acquires vigour: through its means, the brain can no longer command and concentrate towards itself the circulation of the entire system. The preparations of iron are also to be employed to effect this end. You may vary them with the zinc, administering the two on alternate days. The phosphate and carbonate of iron are the best preparations. Small doses long continued are to be used. Large doses may disturb the organs. The sulphate of quinine, in small doses, used as a tonic and not as an anti-periodical, in doses of a quarter or half a grain, is likewise useful. It may be combined with the oil of turpentine, in morbid states of the stomach, accompanied with the voracious appetite of which I have spoken. Upon the whole, however, I decidedly prefer the metallic preparations.

You must never permit constipation of the bowels to take place. To obviate this, rhubarb is an excellent remedy. Avoid the drastic purgatives, which irritate the nervous system, and destroy the digestive powers, which of course is not your object. Open the bowels daily with an injection: this acts upon the lower bowels, which have less connection with the brain, than the upper portion of the intestinal canal. Some of the vegetable tonics as Gentian, Cascarilla, and the tonic anti-spasmodics as Valerian may be commonly resorted to.

The next point in the treatment is to ascertain the seat of the *local affection*. This varies exceedingly. In some instances, I have known a tubercle upon a nerve act as an exciting cause of epilepsy. Again, I have seen it proceed from the point of the finger. Desault cured a case by cutting off the toe. In these cases, a peculiar sensation is felt, commencing at the point of irritation, and ascending apparently along the nerve, until it reaches the brain, when the paroxysm takes place. It has been named aura epileptica. When the aura or sensation goes up slowly from the point of origin, the paroxysm may be often arrested, by applying the tourniquet or a light ligature. Cases are on record, where a piece of glass was the excitant; by cutting it out, the disease terminated. A diseased testicle has acted in the same manner, and relief was afforded by extirpating it. Most frequently, however, some internal abdominal or pelvic visceral affection is the local exciting cause of the disease. This is particularly the case with the uterus, in the female. In the majority of the female epileptic patients, whom I have had under my care, the womb was prolapsed, or enlarged, or carcinomatous, or there was dysmenorrhea from simple nervous irritation of the organ. Unfortunately, after you have completely got rid of the uterine affection, epilepsy will sometimes continue, when the disease has been of long duration. The brain and nervous system have become so much disordered by the long continuance of the disease, that numerous light derangements of the functions are capable of exciting the paroxysms.

Cases of uterine complication, I treat with leeches to the neck of the uterus and to the vulva, cups and blisters to the sacrum, hip baths, in short with the class of remedies, adapted for the relief of dysmenorrhea and uterine irritations. There are cases, in which menstruation is unattended with actual pain, there being simply a sensation of uneasiness or of dragging. Tonics are here of service, particularly the chalybeates. But local depletion, after all, is the remedy upon which you are most to rely. I had a case in this house of a very remarkable character illustrating this view of the subject. The patient labored under maniacal paroxysms, connected with uterine derangement. She was the wife of one of the theatrical orchestral corps, who forced her to travel a week after an accouchement. This brought on first hysteria, and afterwards maniacal excitement with convulsions. These were preceded, as she told me afterwards, by the sensation of a flame of fire rising from the uterus to the brain. The occurrence of the menstrual period aggravated her symptoms. She had been treated, before I saw her, by bleeding and purgatives. I directed the application of leeches to the vulva, a day or two before the menses were expected. They were applied, and the consequence was a severe menorrhagia. I was suddenly sent for, and found her lying on the floor, almost in a state of syncope from loss of blood. A large chamber vessel was filled with blood, and a considerable quantity on the floor of her cell. The application of cold water, arrested the hemorrhage, and the results were most gratifying.

Since that period, the patient has never had another attack; she is now perfectly well, a fine looking, large, fat, healthy woman as you would wish to meet. This case is a fine illustration of the influence of a local affection in determining convulsive paroxysms, and of the advantage of local depletion in relieving and curing them, when dependent on inflammatory irritation. Along with the preceding treatment, leeches or cups along the vertebral column, with setons or caustic issues, according to particular circumstances, are often to be used as adjuvants. Such, gentlemen, is a systematic plan for the treatment of epilepsy, founded on the rationale of the phenomena of the disease. No doubt it will often fail, and you will feel tempted to resort to empiricism. There is an almost endless variety of empirical remedies, many of which I have tried in the course of my practice, but without beneficial results. I will enumerate some of them, as, when a systematic method fails in the treatment of any disease, we must then resort to a tentative practice or empirical remedies.

Equal parts of mustard seed, ginger, and sage, is a remedy for which great success is claimed. I have heard of one or two reputed cures from its use, but it uniformly failed in my hands. The mustard seed produces daily movements of the bowels, the sage exercises a tonic influence, and the ginger is an agreeable stimulant.

Baron Sloet, of Holland, who has great repute for his success in the treatment of epilepsy, has given the following as his remedy. It consists of one pound of the dictamus albus cretensis, or white fraxinella, and of the pulvis zedoaria ℥iss. There are two kinds of the dictamus, one Italian and the other Cretan; Sloet says the Italian is of no use. The dose of the mixture is about two scruples, more or less, according to the case. It is given in the water of linden flowers. Four doses, in bad cases, have been given in a day, but the Zedoary is then reduced to one-half. Immense success is claimed for this prescription. I can say nothing of its value, from my own experience, as I have never been able to obtain the dictamus from any of our apothecaries.

The internal use of lunar caustic as a remedy for epilepsy is now pretty nearly abandoned. I have often administered it here, and never saw it do any good. It is besides exceedingly injurious to the stomach, in the large doses recommended. It is given in doses of from four or five to ten, twenty, and even thirty grains, but I should think it very dangerous in doses of this quantity. I once carried it up to these excessive doses in an old case of epilepsy, in this establishment; shortly afterwards, while I was absent from the city, the patient died, with, it was said, symptoms of inflammation of the stomach, but no post mortem examination was made. There are many fatal cases reported as having occurred in European practice from this cause. I have told you frankly of my own mistake, from a sense of duty; for, in books, you are very seldom warned of the *bad* effects of remedies. Let me then impress it upon you that lunar caustic pushed to high doses, may destroy the mucous membrane of the stomach.

I need not run over any further this list of remedies; I suppose there are at least a thousand of them, and of the most dissimilar characters. I will only add, that, since I have adopted the plan of treating epilepsy, which I have detailed to you, I approach it, if the case be recent, with confidence. The majority of my patients, in private practice, get well; in this house, they are, as you have seen in the history of the patients that were before you, hopeless cases of long standing, which are brought here more for an asylum in their misery, than with any hope of relief.

---

*Saturday, 17th February.*—Dr. Harlan commenced: Gentlemen, the cases of Ophthalmia, exhibited to you at the last lecture, which are not entirely well, are progressing towards a cure. The case of emphysema has terminated fatally. I, at the time, drew your attention to the state of collapse, under which his system laboured, to the coldness of his extremities, blueness of his ears, lips, &c., and other symptoms of want of re-action, pointing to an unfavourable prognosis. His pulse never responded to the stimuli administered, and he died on the third day following the accident which fractured three of his ribs, wounding the lung and producing extensive emphysema. The appearances on dissection, were very similar to those induced in fatal cases of typhous pleurisy. There had been an attempt by nature to establish adhesive inflammation, and some lymph had been formed. The system sinking, serous effusion took place, followed soon by suffocation and death. Like the majority of cases admitted into this hospital, the constitution of this man was enfeebled by intemperate habits. Add to this the prevalence of typhus fever in the wards of the house, and the chances of a favourable termination of such a case as this, are much diminished.

I offer for your consideration to-day a class of diseases, which cannot be too often or too urgently pressed upon your serious attention. The lamentable effects on society, immediately or remotely resulting from the prevalence of Lues venerea are seldom duly appreciated, because seldom presenting themselves at one view. Individual suffering and ruin, social relations disrupted, disease and the seeds of death entailed upon offspring, with a thousand nameless ills, conspire to render this curse of an ill-directed or unrestained instinct, one of the most interesting subjects of research, that can occupy the attention of the surgeon.

Notwithstanding we have good reason to suppose that the Venereal was one of the earliest diseases combated by our profession, the best authorities of our day antagonize each other in the treatment of this malady—at one time, the scrutinizing observations and vast experience of a Hunter were supposed to have settled this important point, scarcely any one doubting the *specific* operation of *mercury* in opposing the operation of the venereal virus, in almost every point. At present the mercurialists and anti-mercurialists in the profession, like the masons and anti-masons of our local politicians, actively oppose each other. Every metropolis in Europe and America furnishes exclusive votaries to either mode of practice; violent prejudices characterize both parties. The French surgeons furnish us with a commendable moderation in this respect, the best authorities having adopted a modified practice as regards the use of mercury in venereal. From their immense hospitals, devoted exclusively to the treatment of this malady, their experience is as extensive as any in the world.

Referring you to the laboured treatises, which have been and are continuing to be published on this subject, and to the University lectures on this important department of surgery, I must confine my remarks, at present, to those practical precepts, which the numerous cases in our wards will enable me to illustrate.

The effects of the inoculation of the venereal virus may be aptly enough denominated,—primary, secondary, and tertiary. The first is merely local, consisting of an ulcer on the genital organs, with or without enlargement of the upper series of the lymphatic glands of the groin. The precise appearances which indubitably characterize a chancre, are at present by no means so satisfactorily ascertained as was once supposed. The ragged edges, ash-coloured surface, and indurated base, are not received as conclusive evidence of true lues by some good authorities, by whom, the constitutional symptoms or affections are considered as alone sufficient to establish the real nature of the disease.

The secondary and tertiary, or constitutional symptoms,—which have all been denominated by some anti-mercurialists, the "Mercurial disease," are characterized by more or less vascular derangement, by irritation, or even inflammatory fevers, inflammation, or ulceration of the fauces, inflammation and pain of the joints and bones, with nodes and caries of the latter; and, finally, by those cuticular affections, included by Hunter under the sweeping appellation of "copper coloured blotches," which are not, however, admitted to implicate various modifications of the disease, requiring peculiar treatment.

We have no case of primary disease, sufficiently recent to demonstrate a well-characterized chancre. This is perhaps of less importance, as you must have had already an opportunity of witnessing this most common form of the malady. Passing on to the secondary form of the disease,—I think the cases before you will at once illustrate at least one very important principle, namely, that mercury can not by any means be justly accused of causing those numerous constitutional or secondary symptoms, which some authors so pertinaciously insist on. Of the numerous cases of *secondary* syphilis, now in our wards, some have supervened where mercury to a greater or less extent had been resorted to in the primary form,—and in others, where not a particle of this mineral had been taken in any form. Some of the patients have been treated by mercurialists, and others by anti-mercurialists.

I do not pretend to deny that mercury may modify when it does not suppress the action of the venereal virus, but I consider it sufficiently proved, that mercury is not necessary to the existence of numerous affections denominated secondary, whilst such affections do never occur, unless the system has been subjected to the action of venereal poison. In delicate females, in children, and in men of undoubted chastity, the system has been, in numberless instances, impregnated with mercury, and there is no instance of secondary syphilitic symptoms having been induced in such cases.

I believe that great improvement in the treatment of venereal has resulted from the discussions on this much mooted point. Some cases doubtless occur, in which this active poison is calculated to produce the most destructive results. The loss of many a nose, of many a soft palate, and of other organs considered of still greater importance, may be justly attributed to the bold and indiscriminate use of mercury in some forms of venereal disease in certain constitutions—but again there occurs other forms of this disease under different circumstances, which are extremely inveterate, if not incurable without its aid. Let me now call your attention more particularly to the cases before you.

---

*Saturday, February 24th.*—At 10 o'clock, Dr. Harlan entered the amphitheatre and commenced: Gentlemen, at my last lecture I made some general remarks upon Syphilis; what I considered to be the most effectual and proper method of treatment, you might have inferred from my commentaries and strictures on the opinions entertained and promulgated by others. I will to-day detail to you the usual course I pursue in this disease, giving it to you without laying claim to any originality.

When a patient in private practice comes to me with venereal, within a few days after it has made its first appearance, I touch the whole surface of the ulcer with caustic potash. This produces very often a good deal of swelling in the organ, with inflammation. I now cover all up in a poultice until the slough separates; dress the sore with mercurial ointment after the slough separates, and heal it up with red-precipitate ointment. In such cases I do not use mercury internally. Where the disease has continued for any time, constitutional symptoms of more or less violence ensue. The lymphatic glands of the groin swell, and you have a tumor in that place, commonly termed a bubo. If possible, I now put the patient to bed, and confine him there for some time. This though you are not always enabled to do, in an affection where most patients deem secrecy of so much importance, and general antiphlogistic measures are pursued. Sometimes, though, the case becomes refractory, and the ulcer will not heal and becomes indolent. In such cases I resort to mercury, provided the patient has not a scrofulous constitution. I give a half or a quarter of a grain of mercury three times a day until the gums become touched.

I seldom or never in my practice have a case of secondary syphilis. I give the mercury just in sufficient quantity to touch slightly the gums and produce its constitutional effects, and in no one case do I produce profuse salivation intentionally. When I first commenced practice it was the custom in this city to make every patient suffering under venereal to spit about a quart a day. Happily this condition of things no longer exists.

I will now call your attention to the subject of the secondary symptoms, which, by the way, when they occur are of primary importance. These are, sore throat, with or without ulceration—nodes, which you know are tumors of the periosteum, disease, or caries of the bones; and the eruptions which appear in the skin. These demal affections were included by Hunter under the denomination of "copper-coloured blotches"—by modern writers these blotches are represented as indicating several important varieties, some to be improved by the use of mercury, others in which this medicine is rather injurious—when the incrustations have been preceded either by papular or postular eruption, and an ulcer is formed beneath the scab—many of the best authorities in Europe recommend abstinence from mercury, and to resort to sarsaparilla, nitric acid, &c. together with such means as improve the general health. As a general practice I think this course a good one, but that exceptions occur, where mercurials produce a speedy improvement in this form of disease, my practice both public and private confirms.

When these "copper-coloured blotches," are attended, on the contrary, with *scaly* eruptions, and the ulcers either do not exist, or are very superficial and disposed to heal—then mercury, is supposed to be most applicable, in secondary syphilis.

The decoction of the woods is in high repute abroad in this stage of the affection. It contains as you know sarsaparilla, mezereon, guiacum, &c. I have tried the decoction of the mezereon bark alone, and found it to answer very well. A compound decoction of sarsaparilla, much used in this house, is prepared by Mr. Marks, the Apothecary.

In some constitutions there is no disposition shown on the part of a venereal ulcer to heal. This probably arises in many cases from the fact of the constitutional forces of the individual being too weak to produce healthy granulations. The destructive process in such cases exceeds vastly the recuperative, and we have, as a consequence, the ill-conditioned, and the phagadenic ulcer; the ravages of the latter, you well know, are at times frightful. I will now show you several specimens of this form of the affection.

[Here several patients were introduced.]

In the phagadenic ulcer, mercury is not admissible; it accelerates the progress of the disease, and hastens often a fatal termination.

In bubo you should always bear in mind the necessity of saving as much skin as is practicable, and for this purpose you should discharge the pus of a suppurating bubo, by means of several small openings, and apply compresses, or linen, or sponges, with pressure sufficient to force out the matter, and produce adhesion between the skin and parts beneath—when no disposition to this adhesion is manifested, stimulating injections, or caustic, must be resorted to—when the skin boundering a venereal ulcer, is flabby, loose, and discoloured, without granulations, it must be removed by the knife or caustic.

Here is a case of hydrocele I operated upon the other day with the acupuncture needle. One needle only was used at the lecture, three more needles were introduced the next day, and allowed to remain 24 hours, and the succeeding four other needles, were similarly resorted to, and on the fifth day, the water had disappeared. What may appear strange to you that not a drop of water followed the withdrawal of the needle. Indeed the surgeon who first proposed the operation states, that only a few drops escaped occasionally in withdrawing the needles.

I will now show a few cases of an affection termed hernia humoralis, but very improperly so called. There is not the slightest affinity between hernia and this disease. The one is a protrusion of intestine, the other an inflammation and swelling of the testicle from gonorrhœa, cold, or external violence, or internal irritation. The modern term of *orchitis* from ορχις, testicle, is much more suitable.

Some persons consider the swelling of the testicle caused by metastasis of the inflammation of the urethra to the gland. This I do not consider by any means correct.

Others again consider that the inflammation in the canal is transmitted directly to the testis through the vasa deferentia. This I cannot think can be the proper explanation; why, if such is the cause, do not both testes become simultaneously affected. This rarely happens. My plan of treatment of orchitis is as follows—bleeding, purgatives, emetics, and sudorifics—in plethoric habits, rest, in a horizontal position, leeching along the cord, warm fomentations and poultices—the most severe and obstinate pain is sometimes removed by the application of a blister on the thigh, over the insertion of the triceps femoris.

Young men who are living very chaste lives, being under matrimonial engagements, are very subject to a swelling of the testes, and will often come to you very much alarmed, and tell you they are fearful they are getting hernia humoralis, at the most critical moment of their lives, assuring you at the same time of their chastity, for some years, &c. Tell them to keep out of the way of venereal excitement and take a purge. It is caused by a swelling of the tubuli sunniferi, from the contained semen. Dionis relates some curious anecdotes connected with this subject.--Vid. Dionis's Surgery.

Hernia humoralis is not necessarily connected with venereal taint. It may arise from blows, and from cold. Some persons attribute it generally to the use of astringent injections in gonorrhæa. John Hunter denied this. Sir Everard Home, Wilson, and others, entertain this opinion. I have never known it to follow the use of sulphate of zinc---this medicine I always use as an injection in gonorrhæa, even in the early stages, and my patients are never troubled either with swelled testicle or stricture. The formula I use is as follows:

℞ Zinci Sulph. ℈i.

Bole Armenian, ʒii.

Aquæ Simp. f℥viii.

The Balsam of Copaiva, as you well know, has long been considered as almost a specific in gonorrhœa. Some years ago, a late Professor of Materia Medica asserted the unfailing virtues of the Balsam to his class. In a few days he had upwards of fifty cases among the students. He plied them all with the balsam; but not one got any better, and he excused himself by saying there was no good Balsam in the city. It is mostly adulterated with spirits of turpentine.

Leucorhoea is the worst disease with which civilized females are subjected, no state or age are exempt from it. Those who lead a sedentary habit are most liable to it, and it sometimes is most obstinate of cure, and produces melancholy effects on the general health. In ordinary cases a wash of a decoction of oak-bark and alum, with the Tinct. Cantharid. internally, will suffice. More difficult cases require the internal use of powerful astringents, as p. alum, p. kino. In old cases, in aged females, or in the dissolute of the sex, inmates of this Hospital, a solution of lunar caustic, five grains to the ounce, has been used as an injection, very successfully.

With some immaterial variations, similar remarks will apply to gleet in men.

A case of stricture was exhibited—the circumstances under which a surgical operation becomes expedient was explained. A modification of Chew's instrument for a division of the stricture when situated beyond the triangular ligament, was shown and commented on—together with some general remarks on the cause, nature, and treatment of stricture of the urethra, terminating the lecture.

