# Erratum in No. 6

**Published:** 1838-03-28

**Authors:** 


### ERRATUM IN NO. 6.

In Dr. Harlan's Clinical Lecture at p. 99, 3d paragraph, 11th line, for "*which are not*," read, "*which are now*."

